# Supplementary material for: Complexome profiling of the Chlamydomonas psb28 mutant reveals TEF5 as an early PSII assembly factor
Source: Plant Cell. 2025 Mar 17;37(6):koaf055. doi: 10.1093/plcell/koaf055 (PMC12164590; doi:10.1093/plcell/koaf055)
Supplement: koaf055_Supplementary_Data [file koaf055_supplementary_data.zip › psb28_interactive.zip!/plots/intensity/qualityPlots/peptides/PCK1_Cre02.g141400.t1.2.html]

### Cre02.g141400.t1.2

Trivial name: PCK1  
SeqCoverage [median]: 9.20  
Mass: 67.54 kDa  
  
 

| Sequence | MissedCleavage | Mass [Da] | wtIntensity | muIntensity | Score |
| --- | --- | --- | --- | --- | --- |
| AIDYLNTLDR | 0 | 1192.61 | 458.30 | 1964.35 | 92.44 |
| ESGLQPR | 0 | 785.40 | 0.00 | 322.87 | 87.58 |
| FGTVLENVDYNPVTR | 0 | 1722.86 | 0.00 | 3261.73 | 126.10 |
| ILTGGPQPIAK | 0 | 1093.65 | 0.00 | 1335.52 | 107.57 |
| ILTGGPQPIAKEDVEK | 1 | 1693.93 | 0.00 | 1879.47 | 46.75 |
| TTLSADPK | 0 | 831.43 | 0.00 | 1519.08 | 121.99 |
|  |  |  | 458.3 | 10283.0 |  |
